# Effects of Acute Exercise and Learning Strategy Implementation on Memory Function

**DOI:** 10.3390/medicina55090568

**Published:** 2019-09-05

**Authors:** Paul D. Loprinzi, Faith Harris, Kyle McRaney, Morgan Chism, Raymond Deming, Timothy Jones, Liye Zou, Miaoqing Tan

**Affiliations:** 1 Exercise & Memory Laboratory, Department of Health, Exercise Science and Recreation Management, The University of Mississippi, Oxford, MS 38677, USA; 2 Exercise & Mental Health Laboratory, School of Psychology, Shenzhen University, Shenzhen 518060, China; 3 Faculty of Athletic Training, Guangzhou Sport University, Guangzhou 510500, China

**Keywords:** cognition, episodic memory, exercise, learning, memory

## Abstract

*Background and Objectives*: Long-term potentiation (LTP), the functional connectivity among neurons, is considered a mechanism of episodic memory. Both acute exercise and learning are thought to influence memory via an LTP-related mechanism. Limited research has evaluated the individual and combined effects of acute exercise and learning strategy implementation (e.g., 3-R technique, cue-integration) on memory, which was the purpose of this study. *Materials and Methods*: For Experiment 1, participants (*n* = 80; M_age_ = 20.9 years) were randomized into one of four experimental groups, including Exercise + Learning (E + L), Learning Only (L), Exercise Only (E), and Control Group (C; no exercise and no learning strategy implementation). The exercise stimulus involved an acute 15-min bout of lower-intensity (60% of heart rate max) walking exercise and the learning strategy involved the implementation of the 3-R technique. Experiment 2 (*n* = 77; M_age_ = 21.1 years) replicated Experiment 1 but addressed limitations (e.g., exposure level of the memory task) from Experiment 1 and employed a higher-intensity bout of exercise (77% of heart rate max). Experiment 3 (*n* = 80; M_age_ = 21.0 years) evaluated these same four experimental conditions but employed a cue-integration learning strategy and a moderate-intensity bout of acute exercise (64% of heart rate max). *Results*: These three experiments demonstrate that both learning techniques were effective in enhancing memory and we also provided evidence of a main effect for acute exercise (Experiment 3). However, we did not observe consistent evidence of a learning by exercise interaction effect. Conclusions: We demonstrate that both acute exercise and different learning techniques are effective in enhancing long-term memory function.

## 1. Introduction

Exercise is important in many aspects of life, including physical and mental health [1]. Additionally, acute exercise has also been shown to have cognitive-enhancing effects [2], including improved memory function [3], with effects observed across the lifespan [4]. As we have thoroughly discussed elsewhere [5,6,7,8,9], there are several mechanisms that may explain this acute exercise–memory relationship, including, for example, exercise-induced long-term potentiation (LTP) [10]. Long-term potentiation, characteristically represented by sustained post-synaptic potentiation, is considered a potential cellular mechanism of episodic memory function [11].

In addition to acute exercise potentially enhancing memory function [4,5,12,13,14,15,16,17], other factors, such as employing learning-based strategies, may help to improve memory performance. As we have detailed elsewhere [18], there are a variety of learning strategies that can be used for improving memory. Of interest to the present study is the utilization of rehearsal and cue-based learning techniques. These specific techniques are evaluated herein as empirical research has been conducted on these strategies and these techniques can be easily adopted by students [19,20]. A rehearsal technique, namely, the 3-R technique [20], involves reading, reciting and reviewing the given memory task before the recall test. This technique is likely to be effective for several reasons. First, after the individual reads the material, they are instructed to recite the material out loud. This may help strengthen the memory trace and provide another cue (auditory) to facilitate memory retrieval. Further, after reciting the material out loud, this may inform the individual of which information they were deficient in remembering, and thus, may deepen encoding during the subsequent re-reading session (i.e., guide their efforts during re-reading). Additionally, the second re-reading provides immediate feedback on the free recall test, which may augment a testing effect. Cues (e.g., identification of a word associated with the target word, e.g., the target word is *marshmallow*, with the cue word being *campfire*), particularly when self-generated, can also effectively enhance memory performance [19]. Effective cues are compatible with the memory in the sense of describing features or attributes of the encoded information. Further, effective cues are those that are distinct, that is, those that have minimal target items subsumed by the cue. These techniques are worthwhile to investigate as they can be easily adopted and implemented among students.

To our knowledge, there are no studies that have conducted a side-by-side comparison as well as evaluated potential combined effects of these strategies (i.e., exercising and implementing a learning strategy) on memory function. This investigation is in direct alignment with recent recommendations [12]. As we have thoroughly discussed elsewhere [8], we anticipate that this will occur from an associativity and/or synaptic tagging and capturing effect. From an associativity perspective, acute exercise may help to stabilize the memory trace via an LTP-related mechanism [8]. Similarly, acute exercise may induce plasticity-related proteins that tag nearby synapses for capturing by the memory stimuli [8]. Relatedly, in the context of a potential additive effect of acute exercise and learning strategy implementation, we speculate that acute exercise will lower the LTP-threshold induced from the learning-strategy implementation. This aligns with other work showing that acute exercise occurring before or during LTP induction is effective in enhancing LTP [21,22]. As we have recently demonstrated in a meta-analysis [23], systematic review [24], narrative review [25], and individual experiments [26,27,28], the timing of acute exercise appears to be critical in enhancing memory function, with acute exercise occurring before memory encoding being effective for young adults. As such, for the present experiments, our acute bout of exercise occurred prior to memory encoding.

For our three experiments, we specifically chose word-list type tasks, as these are commonly used episodic memory tasks [3]. We do see merit in other memory tasks, such as spatial/navigational memory tasks, which may be heavily influenced by neurogenesis [29]. However, we felt that this type of task, via neurogenesis mediation, is likely not applicable given that we employed an acute bout of exercise, as opposed to a chronic exercise protocol. We also do see merit in executive function as an outcome measure, especially since most of the literature on acute exercise and cognition has focused on executive function [2]. However, it is more challenging to implement a learning strategy for an executive function task, as such tasks often do not have a long-duration learning component or a delayed assessment. Episodic memory tasks, however, are well suited for implementing learning strategies. Lastly, as noted herein, we changed to a vocabulary task for Experiment 3 for two reasons. This would then allow us to evaluate a different learning strategy (i.e., cue integration instead of 3-R) and would also easily allow us to increase the difficulty of the task (i.e., increase the number of words). For the 50-word memory task employed in Experiment 3, the 3-R learning technique is less appropriate given the number of words in the task. In addition to alterations in the outcome (memory) assessment across our experiments, we also adjusted the exercise protocol (moderate vs. vigorous exercise). Explanations for these adjustments will be further explained hereafter.

In addition to this being a novel study, this study will contribute to the literature in two unique areas. Firstly, if there is an additive effect of exercise and learning strategy implementation on memory, then this may suggest that these two techniques may have unique or synergistic mechanisms subserving memory, which should spawn the development of additional empirical work on this topic. Secondly, if an additive effect is observed, then this may have important practical and education-related implications, such as recommending the promotion of acute exercise prior to learning [30]. Ultimately, we hypothesize that, individually, employing a learning strategy and acute exercise prior to learning will improve memory function, but we hypothesize that memory will be highest when these are combined (i.e., an additive effect of acute exercise and employing a learning strategy).

## 2. Methods—General

Three experimental studies were conducted, with all data collection occurring in the Exercise and Memory Laboratory at the University of Mississippi. All three studies were approved by the authors’ institutional review board and all participants provided written consent prior to participation.

All three experiments employed a between-subject, randomized controlled design. Across the experiments, participants were randomized into one of four groups, including Exercise + Learning (E + L), Learning Only (L), Exercise Only (E), and Control (C, no exercise and no learning strategy).

The study protocol for these three experiments is shown in Table 1. Across these studies, we varied the learning strategy to increase the generalizability of our findings and allow for adaptations to the difficulty level of the memory tasks (i.e., cue integration technique is more easily implemented for more comprehensive memory tasks). Further, we also varied the intensity of acute exercise, as recent work demonstrates intensity-specific effects of acute exercise on episodic memory [24,31,32]. The first experiment employed a 4-group, between-subject randomized controlled trial. Experiment 1 employed a lower-intensity walking exercise protocol and evaluated whether memory function was influenced by acute exercise coupled with using the 3-R technique; other groups also included only using the 3-R technique, only exercising, and a control group. As addressed hereafter, Experiment 2 was conducted to extend the findings from Experiment 1 by employing a higher-intensity exercise protocol. Lastly, Experiment 3 was also a 4-group, between-subject randomized controlled trial, but instead of utilizing the 3-R technique, participants utilized the cue-integration strategy. 

In general, across the three experiments, we aimed to recruit 80 total participants (20 per group). This is based from a power analysis indicating a sample size of 20 would be needed for sufficient power (d, 0.90; two-tailed α error probability, 0.05; 1-β error probability, 0.80). This was informed from other related work demonstrating relatively large effect sizes (d, 1.04; ƞ^2^_p_ = 0.23–0.29) [14,33]. Further, our employed sample size is similar to other related research that has observed statistically significant effects of acute exercise on memory [26,27].

Recruitment occurred via a convenience-based, non-probability sampling approach (classroom announcement and word-of-mouth). Participants included undergraduate and graduate students between the ages of 18 and 35. To minimize potential confounding effects on memory function, participants were excluded if they: self-reported as a daily smoker [34,35]; self-reported being pregnant [36]; exercised within 5 h of testing [33]; consumed caffeine within 3 h of testing [37]; had a concussion or head trauma within the past 30 days [38]; took marijuana or other illegal drugs within the past 30 days [39]; and were considered a daily alcohol user (>30 drinks/month for women; >60 drinks/month for men) [40].

Across the 3 experiments, statistical significance was established as a nominal alpha of 0.05. Eta-squared (ƞ^2^) was calculated for effect size estimates.

## 3. Methods—Experiment 1

### 3.1. Study Design and Participants

This study employed a 4-arm, parallel group, randomized control trial, involving a 2 (exercise vs. learning) × 2 (immediate vs. delayed memory) factorial design. Eighty (*n* = 80; M_age_ = 21.00, SD = 1.2) years; 61.3% female) young adult participants were recruited. These participants were then randomized into one of four groups (*n* = 20 participants per group) using computer-generated randomization software. This study was approved by the University of Mississippi’s ethics committee (#18-007). All participants provided written consent prior to participation.

### 3.2. Group Assignments

After recruitment, participants were randomly assigned into one of the following four experimental groups, which included: (1) exercising and utilizing a learning strategy, (2) no exercise but does employ a learning strategy, (3) exercising but does not employ a learning strategy, and (4) no exercise and no learning strategy (control group). Specific details on each of these groups are noted in the narrative that follows. Thus, based on this design, we aimed to evaluate whether acute exercise and learning strategy implementation had an independent and/or additive effect on memory function, with the latter involving reading two short passages (paragraphs) and then performing an immediate and delayed memory recall.

- Exercise + Learning; E + L: Participants walked on the treadmill at a brisk pace (explained in detail below) for 15 min. Immediately thereafter, participants sat and rested for two minutes. Following the resting period, participants then applied the 3-R technique to the presented memory task (explained below), and then subsequently, completed the memory task.
- Learning Only; L: Participants did not complete the exercise protocol, instead, they began their protocol by resting for 5 min. Following the resting period, participants then applied the 3-R technique to the presented memory task, and then subsequently completed the memory task.
- Exercise Only; E: Participants walked on the treadmill for 15 min at a brisk pace. After exercising, the participants rested for 2 min and then completed the memory task.
- No Exercise and No Learning; C, control group: Participants rested for 5 min and then completed the memory task. For this 5 min resting condition, participants sat quietly in a wakeful rest state.

### 3.3. Learning Strategy

The 3-R technique stands for Read–Recite–Review. Identical to others [41], those in the 3-R group were told to read the passage (Story A; see Appendix A) once, recite as much as they could remember from the passage out loud, and then read the passage a second time. They then applied this same 3-R technique to Story B (see Appendix A). Following the implementation of this technique, participants were asked to recall the information prior to a 20 min break (short-term memory or STM) and after a 20 min break (long-term memory or LTM). During this break, participants watched a 20 min video of bloopers from Season 2 of the television series “The Office”. This 3-R technique has demonstrated evidence of validity by showing improved immediate- and delayed-free recall when compared to other learning techniques, such as re-reading and note-taking [41].

At the end of the study, participants rated the ease of use of this 3-R technique by answering the following question using a Likert scale (strongly disagree, disagree, neutral, agree, strongly agree): “Using the 3-R technique was easy for me to implement”. Using the same response options, they were asked the following question, “I felt the 3-R technique was useful in helping me remember the passages from the memory tasks”. Lastly, and for comparative purposes, those who exercised were asked the following question at the end of the study, “I felt that the exercise was useful in helping me remember the passages from the memory tasks”.

### 3.4. Memory Task

The memory task was the Logical Memory Test (shown in Appendix A), which has demonstrated evidence of convergent validity with the WMS-IV (Wechsler Memory Scale) logic memory test [42]. This memory task is composed of two short narrative passages (story A and story B) that are 25 lines long. Both stories consist of sequential plots with congruent content similar in length that also feature phrases intended to engage the participant’s emotions. Participants read both stories and then, afterward, were subsequently tested on both stories (immediate and delayed recall). Short-term and long-term memory composite scores were created by summing the results from Story A and Story B together.

In addition to Appendix A showing the two stories, this appendix also displays the scoring rubric for this memory task. While some words are required to receive credit, other lines only require a variation of the phrase or words. Scoring is added up following the participant’s recall of the story, and the higher the score, the better the memory performance of the participant.

### 3.5. Exercise Protocol

Those randomized to the exercise groups (E+L, E) were asked to walk on a treadmill for 15 min at a self-selected “brisk walk” pace. Specifically, we instructed them to self-select a “brisk walking pace, as if they were late to class”. This exact walking protocol has previously been shown to enhance episodic memory performance [27].

Participants walked at a pace of at least 3 miles per hour and maintained their self-selected walking pace during the entire 15 min walking period. A self-selected walking protocol was employed in order to maximize generalizability and because self-selected exercise (vs. imposed intensity) induces a more favorable affective response to exercise [43]. Polar heart rate monitors were used to monitor the heart rates of each participant during the duration of the exercise protocol.

### 3.6. Survey

At the beginning of the visit, to assess mood status, participants completed the Positive and Negative Affect Schedule (PANAS) [44]. For this mood survey, participants rated 20 items (e.g., excited, upset, irritable, attentive) on a Likert scale (1, very slightly or not at all; to 5, extremely). Half of the items constituted a “positive” mood state, with the other half reflecting a “negative” mood state. As a measure of habitual physical activity behavior, and reported as time spent per week in moderate-to-vigorous physical activity (MVPA), participants also completed a survey (Physical Activity Vital Signs Questionnaire) at the beginning of the visit [45]. These assessments were completed to ensure that the groups were similar regarding these parameters, as these parameters could, potentially, influence memory function.

### 3.7. Statistical Analysis

All statistical analyses were computed in SPSS (v. 22, IBM, Armonk, NY, USA). For the memory outcome data, a mixed-measures ANOVA was employed. This involved two between-subject factors, namely exercise (coded as 1 or 0) and learning (coded as 1 or 0). The within-subject factor involved two levels (immediate and delayed memory). Bonferroni-corrected post-hoc t-tests were employed to examine where the differences occurred. For the perceptual outcomes (e.g., whether the participants thought the learning strategy was effective), descriptive statistics (means, SD) were employed.

## 4. Results—Experiment 1

Characteristics of the study participants are shown below in Table 2. Participants, on average, were 21.0 (SD = 1.2) years of age, with all participant characteristics being similar across the four experimental groups.

The physiological responses from the exercise stimulus are shown in Table 3. Across the groups, resting heart rate ranged from 74.7 (14.9) bpm to 76.9 (13.9) bpm, with heart rate increasing up to 119.0 (18.8) bpm at the end of the exercise bout. This achieved exercise intensity was approximately 60% of estimated heart rate max (119 bpm/(220 − 20.7 years)), which falls within the light-intensity exercise range [46].

### 4.1. Memory Results

**Figure 1:**
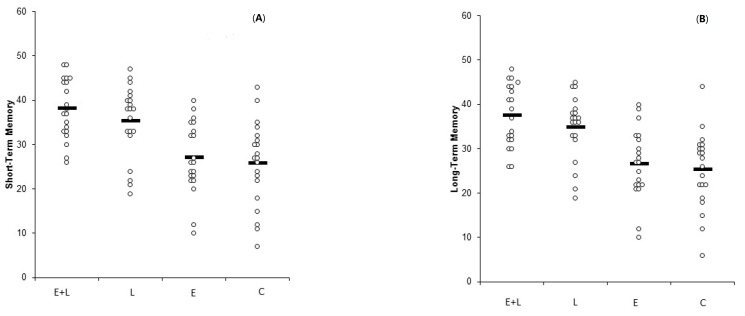
(**A**). Short-term memory scores across the four groups (Experiment 1). (**B**). Long-term memory scores across the four groups (Experiment 1).

The group-level memory scores for each group are shown in Table 4. The individual-level memory results for short-term and long-term memory, respectively, are shown in Figure 1A,B.

In the memory analyses, and for the within-subject effects, there was no main effect for time, F(1, 76) = 3.21, *p* = 0.07, ƞ^2^ = 0.04), time by learning interaction, F(1, 76) = 0.02, *p* = 0.88, ƞ^2^ = 0.0001, time by exercise interaction, F(1, 76) = 0.002, *p* = 0.96, ƞ^2^ = 0.0001, or time by learning by exercise interaction, F(1, 76) = 0.11, *p* = 0.73, ƞ^2^ = 0.002.

For the between-subject effects, there was no main effect for exercise, F(1, 76) = 1.33, *p* = 0.25, ƞ^2^ = 0.01, or learning by exercise interaction, F(1, 76) = 0.16, *p* = 0.68, ƞ^2^ = 0.002, but there was a significant main effect for learning, F(1, 76) = 33.78, *p* < 0.001, ƞ^2^ = 0.31. Post-hoc tests were computed for the learning factor, which demonstrated a significantly greater memory performance among those who received the learning manipulation when compared to those not receiving the learning manipulation (*M*_diff_ = 10.26, SE = 1.76, *t* = 5.81, *p* < 0.001).

### 4.2. Perceptions of Exercise and the Learning Strategy

Among the participants who implemented the 3-R technique, they were asked whether they thought the technique was easy for them to implement. This included response options of strongly disagree (1), disagree (2), neutral (3), agree (4) and strongly agree (5). The mean (SD) score was 4.23 (0.86), suggesting that participants who were randomized into the groups that implemented this technique “agreed” that this technique was easy to implement. Notably, there were no differences between the E+L (*M* = 4.4, SD = 0.94) and L (*M* = 4.1, SD = 0.75) groups, *t* = 1.29, *p* = 0.20, d = 0.41.

These participants were also asked if they felt that this learning technique was useful in helping them remember the content. The mean (SD) for this question was 4.25 (0.70), suggesting that participants “agreed” that this technique helped them remember the content. Notably, there were no differences between the E+L (*M* = 4.4, SD = 0.68) and L (*M* = 4.1, SD = 0.68) groups, *t* = 1.84, *p* = 0.07, d = 0.58.

Lastly, among those randomized into a group involving the acute bout of exercise, they were asked if they felt that the exercise bout was useful in helping them remember the content. The mean (SD) for this question was 3.76 (0.83), suggesting that participants were just under the “agree” threshold regarding their perception that acute exercise helped them remember the content. Notably, there were no differences between the E+L (*M* = 3.9, SD = 0.96) and E (*M* = 3.6, SD = 0.68) groups, *t* = 1.13, *p* = 0.26, d = 0.36.

## 5. Discussion

### 5.1. Experiment 1

Previous studies show that exercise and the use of learning strategies can have a beneficial impact on memory performance individually, but to our knowledge, there are no studies looking at the combined effects of using a learning strategy and engaging in acute exercise on episodic memory function. Our results showed a significant main effect for learning, suggesting a beneficial effect of the 3-R technique on memory function.

Although speculative, perhaps our walking stimulus was not sufficiently intense to substantively alter some of the mechanisms (addressed in the Introduction Section) related to episodic memory function. We initially chose a lower-intensity exercise protocol as this exercise protocol has been shown to enhance memory function in other related research [27]. However, in the context of episodic memory, higher-intensity exercise may be more beneficial [24], particularly when assessing cognition after a break from exercise [2]. Higher-intensity exercise may have a greater effect on enhancing synaptic plasticity in the hippocampus [47,48]. For example, elevated levels of select neurotransmitters can induce various intracellular signaling pathways (e.g., PKA) to facilitate CREB transcription, and in turn, subserve LTP [49]. Thus, to extend the findings from Experiment 1, the exercise protocol for Experiment 2 includes an acute bout of higher-intensity exercise.

In addition to employing a higher-intensity exercise protocol, Experiment 2 also addresses the limitations of Experiment 1. That is, in Experiment 1, the E + L and L groups were exposed to the memory task (Story A and B) twice; for example, they read Story A, then recited Story A out loud, and then re-read Story A. In the other two groups (E and C), they were just exposed to the Story once; that is, they read the story and then recalled as much information as possible. Although this approach allows us to see if memory function is different between E + L and L, as well as between E and C, it makes it challenging to compare, for example, E + L vs. E. This approach (i.e., different time on task) was intentional as it would allow us to confirm that greater exposure to the memory stimuli would be advantageous.

### 5.2. Experiment 2

Our findings from Experiment 1 confirmed that E + L, as well as L, had greater memory performance than E and C. Despite this and based on different exposure characteristics (exposure level to the content), it is not possible to confirm that the greater memory scores were a result of the 3-R technique, or rather, were a consequence of greater exposure to the task. Thus, for Experiment 2, participants in the E and C groups were exposed to the memory task to the same extent as those in E + L and L. That is, those in the E and C groups read the memory stories (A and B) twice. Experiment 2 also evaluated whether a higher-intensity bout of exercise [24] would independently and additively influence memory function.

## 6. Methods—Experiment 2

### 6.1. Study Design and Participants

The sample included 77 young adults (M_age_ = 21.10, SD = 3.3) years; 50.0% female). All other aspects of Experiment 2 were identical to Experiment 1, with the exception of two things. This included (1) employing an acute (15 min) bout of high-intensity exercise (instead of the lower-intensity exercise protocol employed in Experiment 1), and (2) ensuring that all four experimental groups (E + L, L, E, and C) had the same degree of exposure to the memory task (i.e., addressing the limitations from Experiment 1). Details on these two modifications are detailed below. This study was approved by the University of Mississippi’s ethics committee (#19-015). All participants provided written consent prior to participation.

### 6.2. Exercise Protocol

Those randomized to the exercise groups (E + L, E) were asked to jog (high-intensity exercise) on a treadmill for 15 min. Specifically, we aimed to have participants exercise at 70%–85% of their estimated maximum heart rate (using the formula 220 − age) [50]. Heart rate and ratings of perceived exertion were measured at baseline, mid-point (7.5 min), endpoint (15 min), and 10 min post-exercise. After the exercise bout, participants rested (sat quietly) for 10 min and then commenced the memory task.

### 6.3. Group Assignments

Participants were randomly assigned into one of the following four experimental groups.

Exercise + Learning; E + L: Participants jogged on the treadmill for 15 min at 70%–85% of their HR_max_. Immediately thereafter, participants sat and rested for 10 min. Following the resting period, participants then applied the 3-R technique to the presented memory task, and then subsequently completed the memory task. As before, the 3-R task involved reading the story, reciting the story out loud (without looking at the story), and then re-reading the story again.

Learning Only; L: Participants did not complete the exercise protocol, instead, they began their protocol by resting for 10 min. Following the resting period, participants then applied the 3-R technique (identical to E + L) to the presented memory task, and then subsequently completed the memory task.

Exercise Only; E: Participants jogged on the treadmill for 15 min at 70–85% of their HR_max_. After exercising, the participants rested for 10 min and then completed the memory task. This involved reading the story twice, with a short (5 s) pause in between.

No Exercise and No Learning; C, control group: Participants rested for 10 min and then completed the memory task (identical to E; i.e., reading the story twice, with a short (5 s) pause in between).

### 6.4. Statistical Analysis

All statistical analyses were computed in SPSS (v. 22). For the memory outcome data, a mixed-measures ANOVA was employed. This involved two between-subject factors, namely exercise (coded as 1 or 0) and learning (coded as 1 or 0). The within-subject factor involved two levels (immediate and delayed memory). Bonferroni-corrected post-hoc *t*-tests were employed to examine where the differences occurred.

## 7. Results—Experiment 2

Characteristics of the study participants are shown in Table 2. Participants, on average, were 21.1 (SD = 3.3) years of age, with all participant characteristics being similar across the four experimental groups. Demographic characteristics for Experiment 2 were similar to the sample for Experiment 1.

The physiological responses from the exercise stimulus are shown in Table 3. Across the groups, resting heart rate ranged from 76.2 (11.4) bpm to 87.1 (9.9) bpm, with heart rate increasing up to 154.3 (12.6) bpm at the end of the exercise bout. This heart rate of 154.3 bpm for Experiment 2 was significantly higher than the exercise heart rate for Experiment 1 (119.1 bpm) (*p* < 0.001). The achieved exercise intensity for Experiment 2 was approximately 77% of estimated heart rate max (154 bpm/(220 − 20.5 years)), which falls within the vigorous-intensity exercise range [46].

### Memory Results

**Figure 2:**
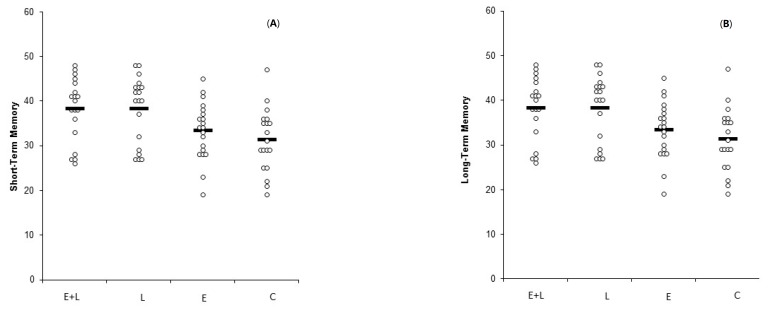
(**A**). Short-term memory scores across the four groups (Experiment 2). (**B**). Long-term memory scores across the four groups (Experiment 2).

The group-level memory scores for each group are shown in Table 4. The individual-level memory results for short-term and long-term memory, respectively, are shown in Figure 2A,B.

In the memory analyses, and for the within-subject effects, there was a main effect for time, F(1, 73) = 30.68, *p* < 0.001, ƞ^2^ = 0.29, but no time by learning interaction, *F*(1, 73) = 0.0001, *p* = 0.99, ƞ^2^ = 0.0001, time by exercise interaction, F(1, 73) = 0.01, *p* = 0.92, ƞ^2^ = 0.0001, or time by learning by exercise interaction, F(1, 73) = 0.42, *p* = 0.51, ƞ^2^ = 0.006. Post-hoc tests were computed for the time factor, which demonstrated a significantly greater memory performance for short-term memory vs. long-term memory (*M*_diff_ = 1.58, SE = 0.28, *t* = 5.65, *p* < 0.001).

For the between-subject effects, there was no main effect for exercise, F(1, 73) = 0.48, *p* = 0.48, ƞ^2^ = 0.007, or the learning by exercise interaction, F(1, 73) = 0.64, *p* = 0.42, ƞ^2^ = 0.007, but there was a significant main effect for learning, F(1, 73) = 14.76, *p* < 0.001, ƞ^2^ = 0.17. Post-hoc tests were computed for the learning factor, which demonstrated a significantly greater memory performance for those who received the learning manipulation when compared to those not receiving the learning manipulation (*M*_diff_ = 5.90, SE = 1.53, *t* = 3.84, *p* < 0.001).

## 8. Discussion—Experiment 2

In Experiment 1, we demonstrated that lower-intensity acute exercise (low- to moderate-intensity) coupled with implementing the 3-R technique had the greatest memory function; however, this group did not statistically differ from the learning-only group. We anticipated that this may be a result of the intensity of exercise, as higher-intensity exercise has recently been shown to more favorably influence episodic memory function [24]. Thus, Experiment 2 replicated Experiment 1, but, rather than employing a low- to moderate-intensity bout of exercise, we employed a high-intensity bout of exercise. Experiment 1 also showed that greater exposure to the memory task improved memory performance, as the E + L and L groups read the story twice, whereas the E and C controls only read it once. Thus, from Experiment 1 it was not possible to conclusively confirm that the 3-R technique itself (i.e., the “reciting” part) was responsible for the improvements in performance. Thus, for Experiment 2, we addressed this limitation by having the E and C groups read the story twice (but not “recite” it aloud). Results from Experiment 2 demonstrated near-identical conclusions when compared to Experiment 1. That is, both E + L and L had the greatest performances in memory, but there were no differences between these two groups. Thus, collectively, results from Experiment 1 and Experiment 2 provide strong evidence favoring the 3-R technique for enhancing memory function. Experiments 1 and 2, however, do not provide evidence that exercise, ranging from low- to high-intensity, independently or additively (with 3-R) influences memory function.

A potential limitation with both Experiment 1 and Experiment 2 is that, possibly, the memory task employed may have made it difficult to observe an additive effect of exercise and the 3-R technique in enhancing memory function. For example, for immediate recall of Story B, participants, on average, recalled 84% (21.1/25) of the content when employing the 3-R technique alone. Thus, although speculative, a ceiling effect may have prevented a potential additive effect (of exercise and the learning technique) from being observed.

## 9. Introduction—Experiment 3

To address this potential ceiling effect issue with the short paragraph-assessed memory task employed in Experiments 1 and 2, for Experiment 3, we re-evaluated this additive four-group paradigm but employed a more challenging memory task. For Experiment 3, we utilized a cue-integrated learning technique to recall a 50-item word list. This learning technique involves being exposed to a list of words and then generating property cue words (i.e., a different word to help remember the target word) to help subsequently recall the target word. Writing down a property word for each target word is a cue because, for the second delayed memory assessment, participants were allowed to use these cue words to help facilitate memory recall.

## 10. Methods—Experiment 3

### 10.1. Study Design

The sample included 80 young adults (M_age_ = 21.04, SD = 1.5), 65.0% female). A four-arm, parallel-group randomized controlled intervention was employed. Participants were randomized into one of four groups, including (1) exercise with cue-integration, (2) cue-integration only, (3) exercise only, and (4) no cue-integration and no exercise (control). The exercise stimulus involved moderate-intensity treadmill exercise for 15 min, while the control group rested (sat). This study was approved by the University of Mississippi’s ethics committee (#18-117). All participants provided written consent prior to participation.

### 10.2. Participants

Each group included 20 participants (*n* = 80). The recruitment approach and eligibility criteria for Experiment 3 was identical to that of Experiments 1 and 2.

### 10.3. Exercise Protocol

The exercise bout involved exercising on a treadmill for 15 min. We aimed to have participants exercise at approximately 70% of their estimated heart rate max (220 − age), representing moderate-intensity exercise [46]. Given that the results between Experiment 1 and Experiment 2 were similar, for Experiment 3, we went back to a lower-intensity protocol, to reduce any potential displeasure associated with higher-intensity exercise.

Immediately after the bout of exercise, participants rested in a seated position for 5 min. After this resting period, they commenced the memory assessment, as described below.

### 10.4. Learning Strategy and Memory Assessment

The learning strategy was modeled after previous work employing a cue-integrated learning technique [19]. Participants viewed 50 words from the Toronto Word Poll. Words were presented one at a time on a computer screen and displayed for 3 s each. Immediately afterward, participants free-recalled as many of the words as possible. Following this free recall:

The control and exercise-only groups re-reviewed the list of words, with each word being presented for 15 s.

The cue-integration-only and exercise with cue-integration groups, however, while viewing each word for 15 s, wrote down one property word for each target word. They were instructed that the property word cannot be a component of the target word (e.g., if the target word is “Primadonna”, the property word cannot be “Madonna” or “Donna”).

After completing this task (i.e., re-reviewing the words for Groups 3–4 or creating property words for Groups 1–2), participants watched a video of “The Office—Bloopers” for 20 min. Immediately following the viewing of this video:

Control and exercise-only groups free-recalled as many words as possible (Delay Recall 1). Immediately after this first delayed recall, they re-recalled as many words as possible (Delay Recall 2).

Cue-integration only and exercise with cue-integration groups also free-recalled as many words as possible (delay recall 1). Immediately after this first delayed recall, they were given their sheet of property words, and while using these cues, they re-recalled as many words as possible (Delay Recall 2).

### 10.5. Statistical Analysis

All statistical analyses were computed in SPSS (v. 22). For the memory outcome data, a mixed-measures ANOVA was employed. This involved two between-subject factors, namely exercise (coded as 1 or 0) and learning (coded as 1 or 0). The within-subject factor involved three levels (immediate, delayed 1 memory, and delayed 2 memory). Bonferroni-corrected post-hoc t-tests were employed to examine where the differences occurred.

## 11. Results—Experiment 3

Table 2 displays the demographic characteristics of the sample for Experiment 3. Participants, on average, were 21.0 (SD = 1.4) years of age, with all participant characteristics being similar across the four experimental groups, with the exception of age (*p* = 0.04). As such, our sensitivity analyses controlled for age. Demographic characteristics for Experiment 3 were similar to the sample for Experiments 1 and 2.

The physiological responses from the exercise stimulus are shown in Table 3. Across the groups, resting heart rate ranged from 74.7 (17.5) bpm to 80.3 (14.4) bpm, with heart rate increasing up to 127.3 (6.6) bpm at the end of the exercise bout. This heart rate of 127.3 bpm for Experiment 3 was slightly higher than the exercise heart rate from Experiment 1 (119.1 bpm). The achieved exercise intensity for Experiment 3 was approximately 64% of the estimated heart rate max (127 bpm/(220 − 20.9 years)), which falls within the moderate-intensity exercise range [46].

### Memory Results

**Figure 3:**
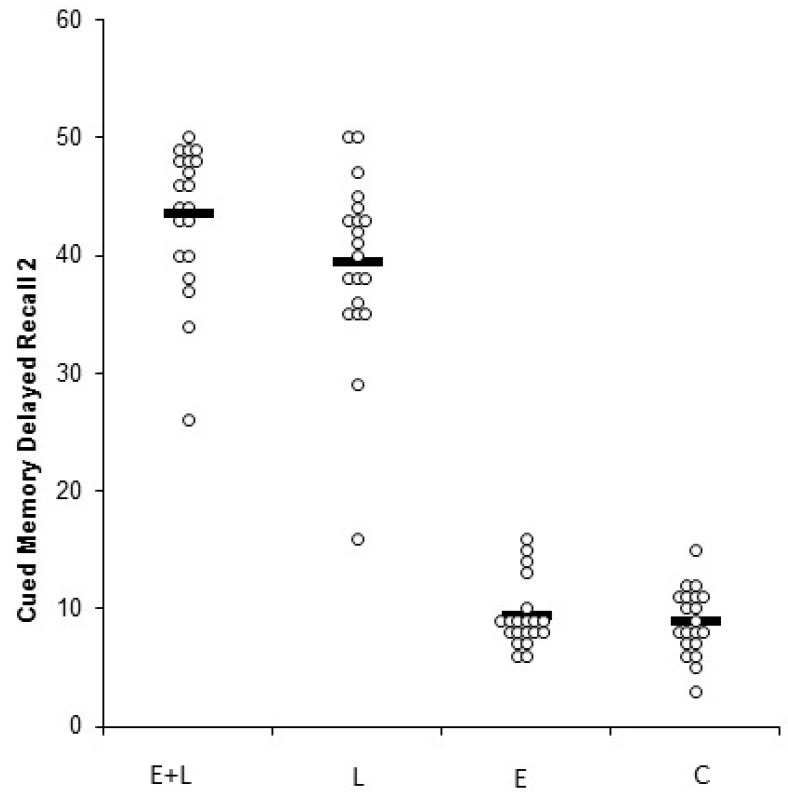
Cued-memory delayed recall 2 across the four experimental groups (Experiment 3).

The memory scores for each group are shown in Table 4. Figure 3 displays the individual and mean scores for delayed 2 memory recall (which involved the utilization of the word cues during memory retrieval) across the four respective groups.

In the memory analyses, and for the within-subject effects, there was a main effect for time, F(2, 152) = 468.5, *p* < 0.001, ƞ^2^ = 0.42), and a time by learning interaction, F(2, 152) = 568.6, *p* < 0.001, ƞ^2^ = 0.51, but no time by exercise interaction, F(2, 152) = 0.70, *p* = 0.49, ƞ^2^ = 0.001, or the time by learning by exercise interaction, F(2, 152) = 1.06, *p* = 0.34, ƞ^2^ = 0.001. Post-hoc tests were computed for the time factor, which demonstrated a significantly lower memory performance for immediate memory when compared to delayed 1 memory (*M*_diff_ = −2.05, SE = 0.57, *t* = 3.55, *p* = 0.002) and delayed 2 memory (*M*_diff_ = −13.47, SE = 1.81; *t* = 7.41, *p* < 0.001), and similarly, delayed 1 memory was significantly lower than delayed 2 memory (*M*_diff_ = −11.43, SE = 1.37, *t* = 8.33, *p* < 0.001).

For the between-subject effects, there was no learning by exercise interaction, F(2, 152) = 1.92, *p* = 0.17, ƞ^2^ = 0.005, but notably, for delayed 1 memory, E + L was greater than L (*M*_diff_ = 3.6, SE = 1.7, *t* = 2.06, *p* = 0.04). We observed a significant main effect for learning, F(2, 152) = 280.6, *p* < 0.001, ƞ^2^ = 0.77, and a significant main effect for exercise, F(2, 152) = 5.03, *p* = 0.02, ƞ^2^ = 0.01. Post-hoc tests were computed for the learning factor, which demonstrated a significantly greater memory performance for those who received the learning manipulation when compared to those not receiving the learning manipulation (*M*_diff_ = 14.38, SE = 0.85, *t* = 16.75, *p* < 0.001). Similarly, post-hoc tests were computed for the exercise factor, which demonstrated a significantly greater memory performance for those who received the exercise manipulation when compared to those not receiving the acute exercise manipulation (*M*_diff_ = 1.92, SE = 0.85, *t* = 2.24, *p* = 0.02).

## 12. Discussion—Experiment 3

Experiment 3, similar to Experiments 1 and 2, demonstrated that the utilized learning strategy was effective in enhancing memory function. For Experiment 3, however, we also observed a significant main effect for acute exercise. This aligns with other research demonstrating that acute moderate-intensity exercise is effective in enhancing memory function [23]. Similar to Experiments 1 and 2, for Experiment 3, we did not observe a statistically significant learning by exercise interaction.

In Experiment 3, we modified the learning strategy from the 3-R technique to a cue-integrated learning strategy. Although it is possible to utilize the 3-R technique for a word-list paradigm, such as that employed in Experiments 1 and 2, for Experiment 3, we specifically wanted to employ a challenging memory task, as we thought that our previous two experiments may have been limited by the relatively easy nature of the memory task. It is very likely that using a 3-R technique would be ineffective for a 50-item word list task. We acknowledge that Experiment 3, thus, deviates from the learning strategy employed in Experiments 1 and 2, but we feel it extends these experiments as the paradigm (acute exercise and learning strategy on memory function) remained the same.

## 13. General Discussion

Overall, our three experiments demonstrate that the 3-R and cue-integration techniques are robust in enhancing short- and long-term memory. We also provide suggestive evidence that acute exercise may also enhance memory function.

Our observations of the beneficial effects of the 3-R technique align with other experiments. For example, the 3-R technique has been shown to enhance the short- and long-term recall of fact-based passages, when compared to re-reading and note-taking strategies [41]. The 3-R technique may also help memory recall of complex passages [41], and the combination of the 3-R technique with meta-comprehension judgements may help to facilitate inference performance on a learning task [20]. Our robust effects of the cue-integration learning technique also align with past research [19]. Greater cue utilization is proportionally related to recall performance and memory performance is more strongly influenced by self-generated cues [19]. Further, utilizing cues that are present during encoding and employing highly specific cues are advantageous techniques [51,52].

In addition to demonstrating that the 3-R technique (Experiments 1 and 2) and cue-integration technique (Experiment 3) robustly influence short- and long-term memory, our results provide suggestive evidence that acute exercise may enhance memory function. This aligns with past research [4,5,12,13,14,15,16,17]. Acute exercise may induce a high action potential, facilitating the potentiation of the memory trace, and thus, induce LTP of the synapses associated with the memory.

The present set of experiments address an important paradigm that has large implications for educational learning. Based on our findings, students should consider adopting the 3-R and cue-integration learning techniques, as they may have large practical implications in enhancing learning and memory. Further, we also demonstrate some evidence that acute, moderate-intensity exercise, may enhance memory function. As such, prior to studying, or prior to attending a lecture, students may benefit by engaging in a brief bout of walking. Importantly, however, future work is needed to identify the most effective learning strategies and whether acute exercise can augment such strategies. This is a ripe area for future research, as continued work is needed to identify the optimal timing, intensity, and duration of exercise within this paradigm. This should be a prioritized area of research given the clear implications this research has for enhancing learning and memory.

Limitations of these experiments include the relatively small homogenous sample, limiting the study’s generalizability. When feasible, future work should employ a larger sample size to minimize concerns with statistical power, which may have influenced our ability to observe statistically significant effects of acute exercise on memory function (for Experiments 1 and 2). Another limitation is that the total experimental protocol time was not the same across all the groups. For example, our control groups did not sit for the same amount of time as the acute bout of exercise. Although this is a limitation, we were concerned that an extended period of sitting would create participant boredom, and depending on the control task (e.g., reading, puzzle) that could be used to minimize this potential boredom, it could, in theory, prime their cognition, or possibly, induce a proactive memory interference effect. Additionally, the researcher who scored the memory task was not blinded to the group assignment of the participant. However, a standardized scoring approach was utilized for all memory assessments. Further, for Experiments 2 and 3, the prescribed exercise intensity was derived from the participant’s estimated maximum heart rate. As such, within each experiment, it is likely that there was some heterogeneity in the exercise intensity zone in which the participants engaged in. Compared to Experiments 2 and 3, in which the exercise intensity was prescribed, for Experiment 1, participants self-selected their walking speed. This inconsistency across all three experiments may reduce the ability to effectively compare our results across the three experiments. Further, we did not employ an objective assessment of the participant’s cardiorespiratory fitness, which could be used to more accurately prescribe the exercise intensity. Strengths, however, include the experimental design, integration of multiple experiments, evaluation of two learning techniques, including both short- and long-term memory assessments, evaluation of different exercise intensities, and evaluation of a topic that has vast practical implications for educational learning.

## 14. Conclusions

In conclusion, our experiments provide strong evidence for the 3-R and cue-integration techniques in enhancing memory function. We also provide some suggestive evidence that acute exercise may enhance memory function. Future work should continue to evaluate whether acute exercise can augment the effects of implementing a learning strategy, but it would be worth evaluating other learning techniques, such as the method of loci and peg-word techniques. Ultimately, the present work, coupled with future work on this topic, may have important education-related implications. Identifying the optimal learning strategy, the optimal acute exercise intensity and duration, and these combinations, may help to enhance learning and memory.

## Figures and Tables

**Table 1:** Study protocol of the three experimental studies.

Group	Start 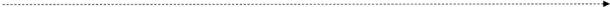 Finish						
	**Experiment 1**						
E + L	15 min moderate-intensity exercise	2 min rest	3-R (read story, recited story, re-read story)	Immediate memory recall	20 min break	Delayed memory recall	
L	5 min rest		3-R (read story, recited story, re-read story)	Immediate memory recall	20 min break	Delayed memory recall	
E	15 min moderate-intensity exercise	2 min rest	Read story once	Immediate memory recall	20 min break	Delayed memory recall	
C	5 min rest		Read story once	Immediate memory recall	20 min break	Delayed memory recall	
	**Experiment 2**						
E + L	15 min high-intensity exercise	10 min rest	3-R (read story, recited story, re-read story)	Immediate memory recall	20 min break	Delayed memory recall	
L	10 min rest		3-R (read story, recited story, re-read story)	Immediate memory recall	20 min break	Delayed memory recall	
E	15 min high-intensity exercise	10 min rest	Read story, 5-s pause, re-read story	Immediate memory recall	20 min break	Delayed memory recall	
C	10 min rest		Read story, 5-s pause, re-read story	Immediate memory recall	20 min break	Delayed memory recall	
	**Experiment 3**						
E + L	15 min moderate-intensity exercise	5 min rest	Encoding of 50 words and then immediate free recall	Re-encoded words and created cue word	20 min break	Delayed 1 recall	Delayed 2 recall with cue
L	5 min rest		Encoding of 50 words and then immediate free recall	Re-encoded words and created cue word	20 min break	Delayed 1 recall	Delayed 2 recall with cue
E	15 min moderate-intensity exercise	5 min rest	Encoding of 50 words and then immediate free recall	Re-encoded words	20 min break	Delayed 1 recall	Delayed 2 recall
C	5 min rest		Encoding of 50 words and then immediate free recall	Re-encoded words	20 min break	Delayed 1 recall	Delayed 2 recall

E + L, Exercise + Learning Group; L, Learning-Only Group; E, Exercise-Only Group; C, Control Group.

**Table 2:** Participant characteristics across the experimental groups.

Variable	Exercise + Learning (E + L)	Learning Only (L)	Exercise Only (E)	Control (C)	*p*-Value
	**Experiment 1**				
*n*	20	20	20	20	
Age, mean years	20.7 (1.3)	21.4 (1.2)	20.7 (1.1)	21.0 (1.3)	0.22
% Female	55.0	65.0	70.0	55.0	0.70
% White	70.0	60.0	65.0	85.0	0.21
BMI, mean kg/m^2^	26.4 (3.7)	25.2 (5.1)	26.8 (5.0)	25.5 (5.9)	0.84
% Right-handed	90.0	100.0	85.0	90.0	0.40
% of participants on medications that affect mood/emotion	10.0	0.0	5.0	15.0	0.31
Physical Activity, mean minutes/week in MVPA	128.4 (257.8)	189.2 (110.6)	129.1 (281.1)	136.5 (103.5)	0.41
PANAS Positive Affect, mean	30.6 (6.8)	28.9 (6.5)	28.2 (7.0)	30.0 (7.2)	0.68
PANAS Negative Affect, mean	14.5 (4.2)	14.7 (4.6)	11.7 (3.1)	14.9 (7.9)	0.19
	**Experiment 2**				
*n*	19	19	20	19	
Age, mean years	20.4 (1.1)	21.8 (1.3)	20.6 (1.0)	21.7 (1.8)	0.45
% Female	68.4	47.4	40.0	52.6	0.33
% White	84.2	63.2	75.0	57.9	0.21
BMI, mean kg/m^2^	26.2 (4.6)	27.0 (4.2)	26.2 (6.0)	26.9 (5.5)	0.95
% Right-handed	84.2	94.7	90.0	78.9	0.59
% of participants on medications that affect mood/emotion	15.8	10.5	10.0	0.0	0.39
Physical Activity, mean minutes/week in MVPA	206.4 (162.5)	194.5 (163.5)	287.0 (307.0)	255.8 (206.0)	0.52
PANAS Positive Affect, mean	24.0 (9.4)	28.5 (8.6)	27.6 (6.5)	28.2 (8.3)	0.26
PANAS Negative Affect, mean	12.8 (5.5)	11.2 (1.8)	12.3 (4.5)	12.4 (2.5)	0.61
	**Experiment 3**				
*n*	20	20	20	20	
Age, mean years	21.3 (1.2)	20.6 (1.0)	20.5 (1.1)	21.6 (2.0)	0.04
% Female	70.0	81.0	57.9	50.0	0.22
% White	65.0	90.5	84.2	85.0	0.23
BMI, mean kg/m^2^	25.8 (5.3)	23.3 (3.6)	26.0 (5.0)	27.2 (5.7)	0.09
% Right-handed	-	-	-	-	
% of participants on medications that affect mood/emotion	10.0	9.5	10.5	0.0	0.54
Physical Activity, mean minutes/week in MVPA	156.0 (125.5)	176.7 (225.9)	171.1 (178.7)	163.8 (98.1)	0.97
PANAS Positive Affect, mean	-	-	-	-	
PANAS Negative Affect, mean	-	-	-	-	

BMI, Body mass index; MVPA, Moderate-to-vigorous physical activity; PANAS, Positive and Negative Affective Schedule. Point estimates are means. Variable estimates (in parentheses) are standard deviations. -, not assessed.

**Table 3:** Exercise-related physiological responses across the experimental groups.

Group	Resting HR	Mid-Point HR (7.5 min)	End-Point HR (15 min)
	**Experiment 1**		
Exercise + Learning (E + L)	76.0 (12.9)	117.0 (17.5)	118.4 (16.7)
Learning Only (L)	76.9 (13.9)	-	-
Exercise Only (E)	74.7 (14.9)	119.0 (18.8)	119.1 (18.4)
Control Group (C)	75.5 (11.0)	-	-
	**Experiment 2**		
Exercise + Learning (E + L)	87.1 (9.9)	145.8 (7.7)	149.1 (8.2)
Learning Only (L)	76.2 (11.4)	76.6 (10.3)	77.7 (11.7)
Exercise Only (E)	77.8 (15.1)	144.8 (6.7)	154.3 (12.6)
Control Group (C)	76.7 (12.2)	77.6 (10.6)	78.0 (10.3)
	**Experiment 3**		
Exercise + Learning (E + L)	80.3 (14.4)	124.8 (9.9)	127.3 (6.6)
Learning Only (L)	78.8 (15.1)	-	-
Exercise Only (E)	77.8 (17.3)	120.0 (12.1)	122.8 (8.7)
Control Group (C)	74.7 (17.5)	-	-

Point estimates are means. Variable estimates (in parentheses) are standard deviations. Notably, for Experiments 1 and 2, the learning technique involved the 3-R technique, whereas for Experiment 3, the learning technique was the cue-integration technique. -, not assessed. HR, heart rate.

**Table 4:** Memory scores across the experimental groups.

Group	Immediate A	Delayed A		Immediate B		Delayed B
	**Experiment 1**					
Exercise + Learning (E + L)	19.2 (3.9)	18.6 (3.8)		18.9 (3.6)		18.9 (3.6)
Learning Only (L)	17.1 (5.2)	16.7 (5.2)		18.2 (3.9)		18.1 (3.2)
Exercise Only (E)	12.7 (4.2)	12.5 (4.5)		14.2 (4.4)		14.1 (4.2)
Control Group (C)	12.4 (5.6)	11.9 (5.2)		13.3 (4.6)		13.3 (4.3)
	**Experiment 2**					
Exercise + Learning (E + L)	18.6 (4.2)	18.1 (4.9)		21.0 (2.4)		20.1 (3.0)
Learning Only (L)	18.7 (4.6)	17.7 (4.9)		21.1 (2.3)		20.4 (3.0)
Exercise Only (E)	16.7 (4.6)	15.4 (4.5)		18.4 (2.8)		17.9 (3.0)
Control Group (C)	15.1 (4.5)	14.1 (4.1)		17.5 (3.5)		17.1 (4.0)
	**Experiment 3**					
	**Immediate Recall**		**Delayed Recall 1**		**Delayed Recall 2**	
Exercise + Learning (E + L)	13.4 (3.4)		20.3 (5.3)		43.5 (6.1)	
Learning Only (L)	11.7 (4.6)		16.7 (5.7)		39.4 (7.6)	
Exercise Only (E)	11.5 (2.7)		9.6 (3.2)		9.4 (2.8)	
Control Group (C)	10.6 (3.6)		8.7 (3.1)		8.9 (2.8)	

Point estimates are means. Variable estimates (in parentheses) are standard deviations. Notably, for Experiments 1 and 2, the learning technique involved the 3-R technique, whereas for Experiment 3, the learning technique was the cue-integration technique.

## Appendix A

**Line**	**Content Encoded**	**Criteria for Scoring**	**Score**	
	**STORY A**			
1	Maria	*Maria* or a variation of	0	1
2	Anderson	*Anderson* required	0	1
3	was a law student	Indication of law student/scholar	0	1
4	at Otago University.	*Otago University* or *University of Dunedin*	0	1
5	She and her two friends	Indication *two other people* joined her	0	1
6	Anna and	*Anna* or variation of name	0	1
7	Michael	*Michael* or variation of name	0	1
8	went skiing	Indication of any snow sport	0	1
9	in Queenstown	*Queenstown* required	0	1
10	over the winter holidays.	Mention of vacation in cold season	0	1
11	They arrived in the afternoon,	Indication of arriving in the afternoon	0	1
12	checked into their hotel,	Indication of arriving at hotel	0	1
13	and went out for dinner.	Indication of going out for meal that night	0	1
14	This night	Indication the situation occurred night of arrival	0	1
15	Maria fell ill	Indication of being sick	0	1
16	with fever,	Indication of abnormally high body temperature	0	1
17	nausea,	Indication of any type of nausea	0	1
18	headache,	Indication of pain or ache in the head	0	1
19	and stomach cramps.	Indication of cramps or pain in the stomach	0	1
20	The doctor advised her	Indication of medical attention/advice	0	1
21	to stay in bed	Indication of advice to remain in bed	0	1
22	for two days	*Two days* required	0	1
23	and to drink tea.	Indication of drinking tea	0	1
24	Maria recovered quickly	Indication of a quick recovery	0	1
25	enjoying the rest of her holiday.	Indication of continuing holiday successfully	0	1
	**STORY B**			
1	Amanda	*Amanda* or variation of name	0	1
2	Wright	*Wright* required	0	1
3	was driving	Indication of driving	0	1
4	to the supermarket in her	Indication of store for destination	0	1
5	blue	*Blue* required	0	1
6	Toyota	*Toyota* required	0	1
7	along Church Road,	*Church Road* or *Street* required	0	1
8	when she saw	Indication of seeing	0	1
9	a white	*White* required	0	1
10	limousine.	Indication of stretch/long vehicle	0	1
11	She was excited	Indication of aroused emotional state	0	1
12	thinking this may be a celebrity’s car	Indication of famous person	0	1
13	visiting her town	Indication of visiting temporarily	0	1
14	for a concert.	Indication of performing	0	1
15	She slower down	Indication of slowing down her car	0	1
16	and tried to get a closer look.	Indication of trying to see famous person	0	1
17	Just then	Indication of a second event at the same time	0	1
18	her two	*Two* required	0	1
19	young	Indication of youth	0	1
20	children,	Indication of children	0	1
21	sitting in their back seats,	Indication of back seat/back of car	0	1
22	started quarrelling.	Indication of argument or being loud	0	1
23	She told them	Indication she spoke to her children	0	1
24	to be quiet,	Indication she asked them to behave	0	1
25	and continued her trip.	Indication she continued the trip	0	1
